# Prevalence and implications of significance testing for baseline covariate imbalance in randomised cancer clinical trials: The Table 1 Fallacy

**DOI:** 10.1016/j.ejca.2023.113357

**Published:** 2023-09-22

**Authors:** Alexander D. Sherry, Pavlos Msaouel, Zachary R. McCaw, Joseph Abi Jaoude, Eric J. Hsu, Ramez Kouzy, Roshal Patel, Yumeng Yang, Timothy A. Lin, Cullen M. Taniguchi, Claus Rödel, Emmanouil Fokas, Chad Tang, Clifton David Fuller, Bruce Minsky, Tomer Meirson, Ryan Sun, Ethan B. Ludmir

**Affiliations:** a Department of Radiation Oncology, Division of Radiation Oncology, The University of Texas MD Anderson Cancer Center, Houston, TX, USA; b Department of Genitourinary Medical Oncology, Division of Cancer Medicine, The University of Texas MD Anderson Cancer Center, Houston, TX, USA; c Department of Translational Molecular Pathology, Division of Pathology/Lab Medicine, The University of Texas MD Anderson Cancer Center, Houston, TX, USA; d Insitro, South San Francisco, CA, USA; e Department of Biostatistics, University of North Carolina at Chapel Hill, Chapel Hill, NC, USA; f Department of Radiation Oncology, Stanford University, Stanford, CA, USA; g Department of Radiation Oncology, University of Texas Southwestern Medical Center, Dallas, TX, USA; h Department of Radiation Oncology, Memorial Sloan-Kettering Cancer Center, New York, NY, USA; i Department of Biomedical Informatics, The University of Texas Health Science Center at Houston, Houston, TX, USA; j Department of Radiation Oncology and Molecular Radiation Sciences, Johns Hopkins University School of Medicine, Baltimore, MD, USA; k Department of Gastrointestinal Radiation Oncology, Division of Radiation Oncology, The University of Texas MD Anderson Cancer Center, Houston, TX, USA; l Department of Experimental Radiation Oncology, Division of Radiation Oncology, The University of Texas MD Anderson Cancer Center, Houston, TX, USA; m Department of Radiotherapy and Oncology, University of Frankfurt, Frankfurt, Germany; n Frankfurt Cancer Institute, Frankfurt, Germany; o German Cancer Research Center (DKFZ), Heidelberg, German Cancer Consortium (DKTK), Partner Site Frankfurt am Main, Frankfurt, Germany; p Department of Genitourinary Radiation Oncology, Division of Radiation Oncology, The University of Texas MD Anderson Cancer Center, Houston, TX, USA; q Davidoff Cancer Center, Rabin Medical Center, Petach Tikva, Israel; r Department of Biostatistics, The University of Texas MD Anderson Cancer Center, Houston, TX, USA

**Keywords:** Table 1 Fallacy, Testing for baseline differences, Covariate imbalance, Randomised controlled trials, Significance testing for baseline characteristics, Oncology, Phase III

## Abstract

**Background:**

The ‘Table 1 Fallacy’ refers to the unsound use of significance testing for comparing the distributions of baseline variables between randomised groups to draw erroneous conclusions about balance or imbalance. We performed a cross-sectional study of the Table 1 Fallacy in phase III oncology trials.

**Methods:**

From ClinicalTrials.gov, 1877 randomised trials were screened. Multivariable logistic regressions evaluated predictors of the Table 1 Fallacy.

**Results:**

A total of 765 randomised controlled trials involving 553,405 patients were analysed. The Table 1 Fallacy was observed in 25% of trials (188 of 765), with 3% of comparisons deemed significant (59 of 2353), approximating the typical 5% type I error assertion probability. Application of trial-level multiplicity corrections reduced the rate of significant findings to 0.3% (six of 2345 tests). Factors associated with lower odds of the Table 1 Fallacy included industry sponsorship (adjusted odds ratio [aOR] 0.29, 95% confidence interval [CI] 0.18–0.47; multiplicity-corrected *P* < 0.0001), larger trial size (≥795 versus < 280 patients; aOR 0.32, 95% CI 0.19–0.53; multiplicity-corrected *P* = 0.0008), and publication in a European versus American journal (aOR 0.06, 95% CI 0.03–0.13; multiplicity-corrected *P* < 0.0001).

**Conclusions:**

This study highlights the persistence of the Table 1 Fallacy in contemporary oncology randomised controlled trials, with one of every four trials testing for baseline differences after randomisation. Significance testing is a suboptimal method for identifying unsound randomisation procedures and may encourage misleading inferences. Journal-level enforcement is a possible strategy to help mitigate this fallacy.

## 1. Introduction

Significance testing is often used in randomised controlled trials to assess for imbalances in the baseline demographic and clinical characteristics among randomised participants. However, this practice, known as the ‘Table 1 Fallacy,’ has faced significant criticism since the seminal work of K.J. Rothman discussing this concern in 1977 [1,2]. The randomisation process, when performed appropriately, effectively prevents systematic biases in confounding baseline characteristics between groups by randomly allocating treatments within the different categories of each covariate [3]. Thus, randomisation provides a clear advantage for inferring treatment effects compared to non-randomised studies, which lack capability of fully adjusting for biases affecting treatment selection. While randomisation helps prevent systematic differences in baseline variables in this way, randomisation does not guarantee balance between groups, as differences due to chance may still occur randomly, particularly in small pilot trials or in cluster trials with small numbers of groups [4]. However, unlike systematic biases, chance imbalances do not threaten inference validity; in fact, it is a key underlying assumption of the conventional analysis of randomised trials that multiple known and unknown confounders are unbalanced by chance [5]. Consequently, engaging in significance testing of baseline variable distributions after sound randomisation is not informative, since any baseline variable differences following unbiased randomisation are already known to be due to chance; in other words, significant findings are, by definition, false positives [6,7]. The importance of imbalances occurring due to chance, therefore, should not be determined based on significance tests, but rather, on clinical reasoning and impact on study outcomes [8].

Despite discouragement from the CONSORT guidelines, along with other research entities and the statistical literature, the extent to which the Table 1 Fallacy persists in modern randomised controlled trials remains unknown [7,9–14]. Thus, this study aimed to investigate the frequency of the Table 1 Fallacy by analysing a large-scale database of contemporary phase III randomised controlled trials in oncology [15]. This study also sought to identify factors associated with the practice of baseline variable significance testing.

## 2. Methods

### 2.1. Study design and participants

This cross-sectional analysis focused on phase III randomised controlled trials in the field of oncology registered on ClinicalTrials.gov. Eligible trials were identified by using an advanced query that incorporated the search terms ‘cancer,’ phase ‘Phase 3’, study results ‘With Results,’ and status ‘excluded: Not yet recruiting.’ Exclusion criteria encompassed non-randomised trials, single-arm studies, phase I or II studies, cancer prevention trials, unpublished trials, trials published in abstract form only, and trials lacking a baseline characteristics table (Fig. S1). Trials with publications through 2020 were included. Since trial-level data were publicly available, institutional review board approval was not required. This study adhered to the Strengthening the Reporting of Observational Studies in Epidemiology (STROBE) reporting guidelines [16].

### 2.2. Objectives

The primary objective involved assessing the frequency of significance testing used to compare baseline characteristics among participants in contemporary phase III oncology trials. Secondary objectives aimed to explore associations between the presence of baseline significance testing and trial characteristics. Relevant information about the trials was extracted from the trial registry, protocols, and/or primary publication, which included the number of baseline variables, the number of baseline significant tests, the findings of baseline significance testing and their interpretation by the trial authors, and the use of multiplicity corrections for baseline significance testing. Additionally, journals publishing at least five trials were defined as either European or American, given the different frequency of the Table 1 Fallacy between European and American journals found in the analysis of Austin et al (Table S1) [17]. Journals were excluded from this categorisation if they did not have a clearly defined locale or were not clearly representative of or sponsored by a medical society with a well-defined locale.

### 2.3. Statistical analysis

Trends in significance testing over time were examined through ordinary least squares linear regression, where the slope (m) of the regression represented the rate of change. For trials containing the Table 1 Fallacy without multiplicity corrections, the number of significance tests for baseline differences was recorded for each trial and then used to apply Bonferroni corrections at the trial level to the reported *P* value to assess the impact of multiplicity correction on the rate of statistically significant findings. The relationship between significance testing and trial factors was assessed by both unadjusted and adjusted binary logistic regression. To avoid conditioning variables that would worsen bias of the adjusted logistic regression, structural causal models were created for each factor by using DAGitty to identify confounder variables on directed acyclic graphs [18–20]. After input of each factor onto the DAGitty interface, causal directionality for each variable relationship was drawn according to the most plausible causal pathway. With the outcome definition fixed as the Table 1 Fallacy, each variable was sequentially selected as the primary factor of interest, and the identification of other variables as either confounders or non-confounding ancestors was recorded for each individual factor-outcome relationship, as characterised by DAGitty and confirmed by the principles of directed acylic graphs [21]. A multivariable binary logistic regression model for each factor of interest, adjusting for confounders unique to that factor, examined the effects of the factor of interest on the odds of the Table 1 Fallacy using adjusted odds ratios (aORs) after elimination of variables exhibiting multicollinearity by Lasso selection. Notably, the association between a factor of interest and the Table 1 Fallacy should be interpreted based on the effect estimate for that factor’s specific multivariable confounder-adjusted model. Interpreting the effect estimates of confounders that are present in the model as representative of their association with the outcome or ‘mutually adjusted’ is known as the Table 2 Fallacy and should be avoided [22]. To account for multiple comparisons, Bonferroni corrections were applied to the secondary objective analyses (correlation between trial factors and the Table 1 Fallacy). Any multiplicity-corrected *P* calculated as greater than 1.00 was set to 1.00. The significance level (α) after multiplicity correction was set at 0.05. All statistical tests were two-sided, and 95% confidence intervals (CIs) were reported. All analyses were conducted with SAS v9 (Cary, NC) and plots were generated by Prism v9 (GraphPad, La Jolla, CA).

## 3. Results

A total of 1877 trials from ClinicalTrials.gov were initially screened, and 765 trials met the eligibility criteria for analysis (Fig. S1). These trials, published between 2002 and 2020, enroled a combined total of 553,405 patients. The baseline variables tables in the analysed trials reported a median 11 variables (interquartile range [IQR] 9–15, range 3–68).

**Fig. 1.**
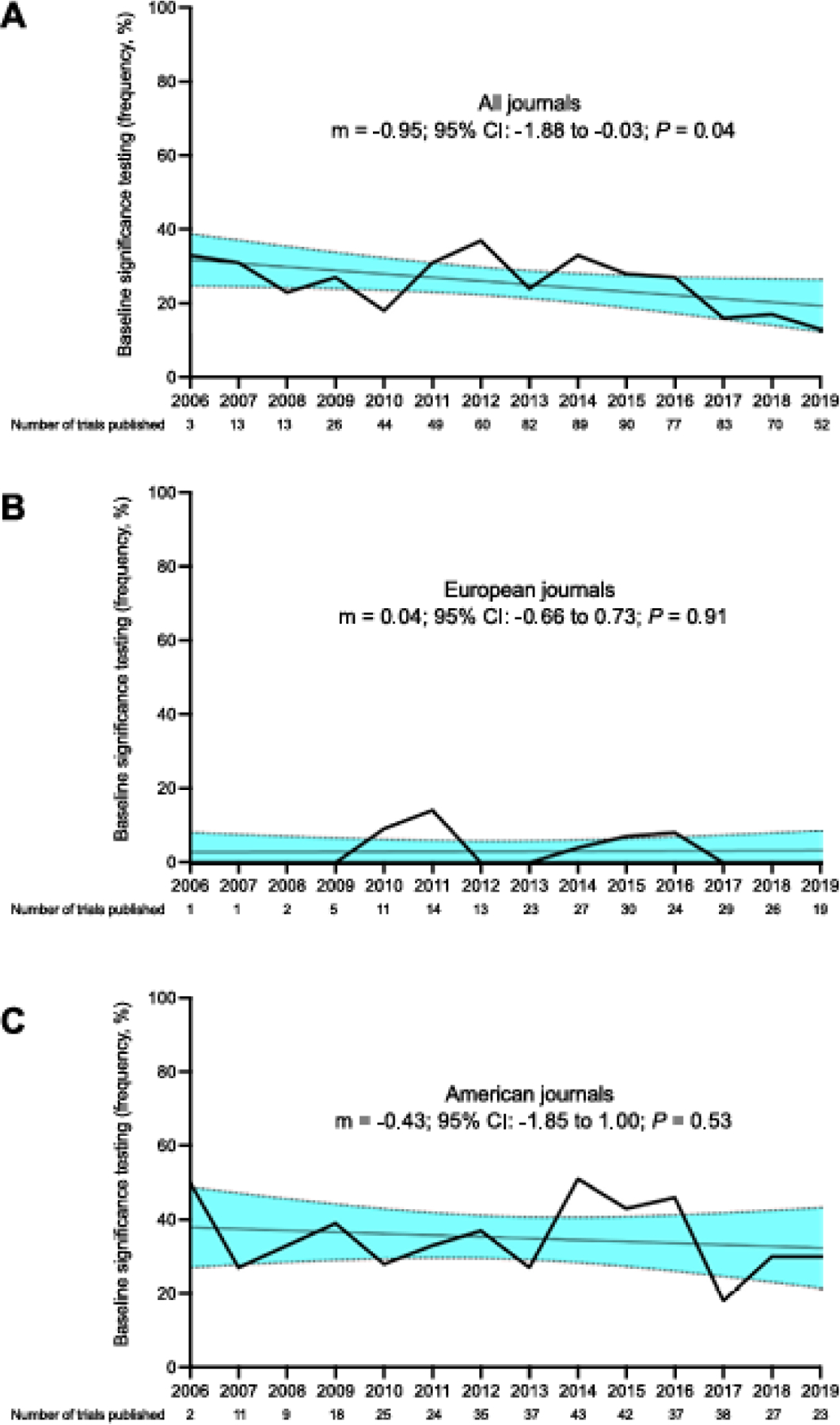
The rate of significance testing for baseline variables over time according to publication year. The linear regression over time is shown with the shaded regions representing the 95% confidence interval of the slope (m). (A) All studied trials. (B) Trials published in journals defined as European. (C) Trials published in journals defined as American.

Among the 765 trials, 188 trials (25%) used significance testing to assess the balance of baseline characteristics after randomisation (Table 1). The rate of trials that included baseline significance testing declined over time during the study period (m = −0.95; 95% CI: −1.88 to −0.03; *P* = 0.04) (Fig. 1A). A median of 10 statistical tests comparing baseline variables were performed per trial (IQR 8–15, range 1–68), resulting in a total of 2353 baseline variable significance tests performed in aggregate. Among the 2353 tests performed, 59 tests (3%) were interpreted as evidence of statistically significant differences according to the trial investigators. The number of baseline variables reported was not significantly different (*P* = 0.26, multiplicity-corrected *P* = 1.00) between trials with the Table 1 Fallacy (median 10, IQR 8–15) and without the Table 1 Fallacy (median 11, IQR 9–15). However, conducting larger numbers of tests was correlated with both an increased odds of finding a statistically significant result (OR 1.07; 95% CI 1.02–1.11; *P* = 0.003) and increased number of statistically significant results (linear regression coefficient [expected increase in the number of significant baseline tests per each additional baseline test performed] β = 0.02; 95% CI 0.008–0.03; *P* = 0.0008). Of the 44 trials reporting statistically significant differences in baseline variables, only one trial (2%) attempted to address this finding in their subsequent analysis, whereas the other trials (98%) reported the finding without further discussion of its implications on the study outcomes or relationship to chance.

None of the 188 trials that used significance testing for baseline variable differences accounted for multiplicity. Of the 44 trials showing statistically significant findings, 36 trials reported specific *P* values, whereas 8 trials reported ranges without specific values (e.g. *P* < 0.05). Application of individual-trial–level multiplicity corrections with the Bonferroni method to 51 *P* values reported as significant by the trial authors eliminated 45 significant findings (88%) using the same definition of α, leaving six differences that remained statistically significant. The overall aggregate rate of significant differences after applying Bonferroni multiplicity corrections was 0.3% (six of 2345).

The characteristics of trials with or without baseline significance testing are presented in Table 2 along with their univariable correlations with the Table 1 Fallacy. Trials investigating metastatic solid tumours showed a lower frequency of baseline significance testing compared with trials of haematologic malignancies (77 of 413 [19%] versus 47 of 148 [32%], unadjusted OR 0.49; 95% CI: 0.32–0.75; *P* = 0.001, multiplicity-corrected *P* = 0.04). Studies evaluating supportive care demonstrated higher use of baseline significance testing compared with medical therapy (57 of 131 [43%] versus 122 of 608 [20%], unadjusted OR 3.07; 95% CI: 2.06–4.75; *P* < 0.0001, multiplicity-corrected *P* < 0.0001). Industry-sponsored trials were less likely to use baseline significance testing (103 of 593 [17%] versus 85 of 172 [49%], unadjusted OR 0.22; 95% CI: 0.15–0.31; *P* < 0.0001, multiplicity-corrected *P* < 0.0001). Furthermore, the Table 1 Fallacy was observed less often for trials enroling more patients (≥795 patients versus < 280 patients: unadjusted OR 0.25; 95% CI: 0.15–0.41; *P* < 0.0001, multiplicity-corrected *P* < 0.0001). Rates of baseline significance testing appeared to vary according to the publication journal (Tables S2). With journals categorised according to locale, 227 trials were published in European journals and 378 trials were published in American journals (with the remaining 160 trials published in journals that did not have a clearly defined locale or were not clearly representative of a medical society with a well-defined locale). Trials published in a European journal were less likely to use baseline significance testing compared with trials published in an American journal (eight of 227 [4%] versus 132 of 378 [35%], unadjusted OR 0.07; 95% CI: 0.03–0.14; *P* < 0.0001, multiplicity-corrected *P* < 0.0001). On subset analysis based on journal locale, there appeared to be no strong change over time in the rate of the Table 1 Fallacy for either European journals (m = 0.04; 95% CI: −0.66–0.73; *P* = 0.91) or American journals (m = −0.43; 95% CI: −1.85 to 1.00; *P* = 0.53) (Fig. 1B–C). As of May 24, 2023, a specific policy recommendation against the Table 1 Fallacy beyond simple reference to CONSORT guidelines was found in 29% of journals (five of 17) which had published a minimum of five trials examined in this study. Of these, a policy was found in 2/4 journals defined as European and 3/7 journals defined as American.

**Fig. 2.**
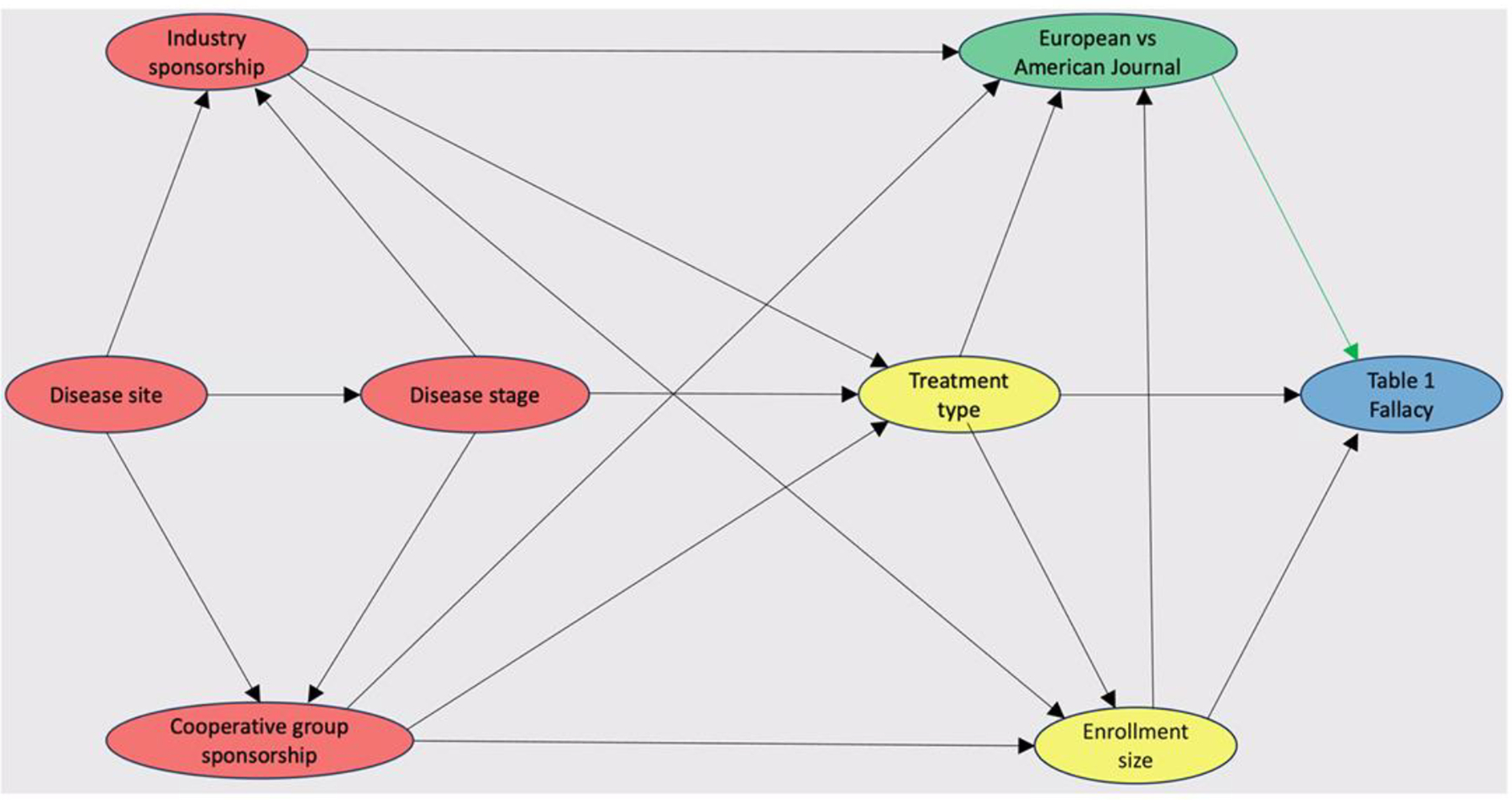
Structural causal model of the relationships between the journal locale, confounding variables, and the Table 1 Fallacy. A green rectangle indicates the exposure of interest (European versus American journal), and a blue rectangle represents the outcome of interest (Table 1 Fallacy). Yellow rectangles indicate confounders, and red rectangles indicate a non-confounding ancestor of both exposure and outcome. The green arrow represents the causal path, and the black arrows represent biasing paths.

Structural causal models were created for each trial factor and plotted on directed acyclic graphs to identify confounders. The relationships of trial characteristics with journal locale and with industry sponsorship are reported in Tables S3–S4. An increased proportion of trials published in American journals were sponsored by cooperative groups relative to European journals, and likewise an increased proportion of trials published in European journals were sponsored by industry versus American journals. Industry-sponsored trials appeared to have an increased proportion of solid metastatic tumour trials, medical therapy trials as opposed to supportive care or local therapy, and lack of cooperative group sponsors compared with trials that were not funded by industry. The full results of entry of structural causal model–informed factor-unique multivariable regression models with Lasso selection to reduce multicollinearity are reported in Tables S5–S11. An example directed acyclic graph is shown in Fig. 2 identifying enrolment size and treatment type as confounders for the effect of journal locale (European versus American) on the Table 1 Fallacy. After confounder and multicollinearity adjustment, three factors remained associated with reduced odds of the Table 1 Fallacy (Table 3). Among all models, publication in a European journal had the strongest association with reduced odds of the Table 1 Fallacy (ref American journal, aOR 0.06; 95% CI 0.03–0.13; *P* < 0.0001, multiplicity-corrected *P* < 0.0001) (Table S5). Trials with larger numbers of patients were also associated with reduced odds of the Table 1 Fallacy (≥795 versus < 280 patients: aOR 0.32; 95% CI 0.19–0.53; *P* < 0.0001, multiplicity-corrected *P* = 0.0008) (Table S6) as well as trials with industry sponsorship (aOR 0.29; 95% CI 0.18–0.47; *P* < 0.0001, multiplicity-corrected *P* < 0.0001) (Table S7).

## 4. Discussion

This study provides a comprehensive analysis of the contemporary randomised controlled trial oncology literature and highlights the persistent and prevalent practice of significance testing for baseline differences in modern oncology trials. Despite the implementation of rigorous trial quality assurance procedures, protocol registration, and pre-specified statistical analysis plans, as well as numerous papers and editorials on the Table 1 Fallacy, the incidence of testing for baseline differences remains high in modern cancer research [14,23]. Specifically, our findings reveal that approximately one out of every four phase III cancer trials continues to include this methodological concern [24]. The high prevalence of baseline significance testing emphasises the need for increased awareness and adherence to CONSORT guidelines by trial authors, editors, and peer reviewers.

The implications of conducting significance testing for post-randomisation baseline differences are far-reaching and have important implications for the current and future landscape of clinical trial research. The consistent and widespread occurrence of this statistical fallacy suggests a gap in the understanding and application of significance testing. The presentation of *P* values in clinical trials is often done within the context of hypothesis testing. Oncology randomised controlled trials are not carried out to test the hypothesis that baseline variables are different between randomised groups. Therefore, including *P* values for baseline differences may misleadingly imply that comparing for these differences is part of the study hypothesis. Not only can this practice misinform readers about the balance of randomised groups, but it can also lead to erroneous interpretations of the study’s findings.

Despite the publication of numerous editorials by statisticians and journal editors advising against testing for baseline differences, evidence on the prevalence of the practice has been limited [8]. A seminal publication in 1990 brought attention to the issue by revealing that 58% of randomised controlled trials (46 of 80 trials) published in four prominent journals from 1987 to 1988 included baseline variable significance testing [25]. One decade later, another study reproduced those findings, with approximately 50% of sampled randomised controlled trials published in 1997 still containing the Table 1 Fallacy [26]. Subsequent analysis conducted in 2007 revealed a modest decline in the Table 1 Fallacy incidence to 38%, with other series showing rates of 39% in the dental literature and 62% in the orthopaedic literature [17,27,28]. Interestingly, our current study implies that testing for baseline imbalance may be less common in oncology than in other fields. Furthermore, the decline we observed in the rate of baseline variable testing from 2006 through 2019 aligns with the overall gradual decline since publication of the seminal study by Altman et al. in 1990, supported by the analyses of Assman et al. in 2000 and Austin et al. in 2010 [17,25,26].

It has been proposed that testing for baseline differences may yield insights into unsound randomisations, which, for example, may arise due to failure of the random number generator or fraud [29–31]. While the select use of these tests as a forensic tool to detect subverted allocation procedures can have some utility, their use in Table 1 should not be standard, particularly given the risk of false positive findings [32–34]. Our study revealed that only 3% of the statistical tests conducted for baseline variable differences in this large sample size yielded significant results. Unsurprisingly, this rate aligns closely with the type I error assertion probability α set at 0.05, which strengthens the argument against this alternative utility for baseline testing [6]. Others may assert that the use of multiplicity corrections could reduce the likelihood of type I errors associated with the multiple tests inherent to baseline covariate comparisons. However, our data also show that the application of multiplicity corrections resulted in a reduction of statistically significant findings to a mere 0.3% of all tests. Of note, trials may be more balanced than what would be expected of simple randomisation if they used randomised blocks or other balancing procedures, or if there is a time trend in covariates. This would tend to reduce the type I error rate. Importantly, our findings highlight the lack of value baseline significance testing holds for identifying improper randomisation procedures, as multiplicity-corrected significant *P* values are rare, and moreover, significant or non-significant *P* values do not signify or confirm appropriate or inappropriate randomisation methods [29]. Therefore, in consideration of these findings, and recognising that a prevailing inclination exists among physicians and readers to interpret *P* values dichotomously without considering the potential for type I and type II errors and to confuse them with posterior probabilities (i.e. regarding *P* < 0.05 as indicating true difference and *P* > 0.05 as indicating true similarity), our findings provide a valuable impetus against using significance testing to assess for sound randomisation procedures [35].

Another counterargument occasionally used for baseline significance testing is that, even if the randomisation procedure is sound, significance testing may detect baseline imbalances occurring due to chance, which can then be accounted for in subsequent inferential analysis. This argument is problematic for several reasons. First, as sample size increases, such as in larger phase III trials, random differences between groups are expected to decrease, but the value of accounting for the effect of these imbalances on outcome heterogeneity remains [3,5,36]. Second, chance imbalances do not prevent valid inferences from a randomised trial, and a fundamental underlying assumption of the conventional analysis of randomised trials is that there are chance imbalances in both measured and unmeasured confounders [5]. Third and perhaps most importantly, chance imbalances meeting statistical significance at α of 0.05 do not necessarily constitute clinically meaningful imbalances.

Rather than statistical testing for baseline differences, one recommended strategy is to inspect the between-group difference or ratio of baseline summary statistics for each confounding baseline variable, and conduct sensitivity analyses in cases where that difference may have a material impact on the trial’s conclusions, since the true question of interest is not whether the covariate distribution differs between groups, but rather, whether conditioning on the covariate would alter a conclusion [3]. Ideally, for baseline variables known a priori to have strong prognostic effects on the outcome of interest, approaches to account for such effects, such as stratification or adjustment in regression models, should be considered at the time of trial design rather than after a post-hoc inspection process [5,37]. Furthermore, irrespective of whether strongly prognostic variables are balanced between groups following randomisation, the omission of these variables from the regression can increase bias in the treatment estimates [5,38,39]. Thus, analyses adjusting for strongly prognostic baseline factors, regardless of whether chance imbalances may or may not occur in these covariates, should be considered at the time of trial design [5].

The results of this study provide insights into several factors associated with lower odds of the Table 1 Fallacy. Possible factors such as the strict adherence to standardised industry guidelines may have contributed to reduced incidence of the Table 1 Fallacy among industry-funded trials [37,40]. Trials enroling larger numbers of patients were also associated with a lower odds of the Table 1 Fallacy, which may reflect the funding and statistical resources available to larger trials. Among all models, however, the factor associated with lowest odds of the Table 1 Fallacy was publication in a European journal, similar to the previous findings of Austin et al., compared with publication in an American journal [17]. Moreover, there appeared to be no decrease in the rate of the Table 1 Fallacy among American journals over time. As of May 24, 2023, a specific policy recommendation against the Table 1 Fallacy was found in 29% of journals (five of 17) which had published at least five trials examined in this study, although extrapolating the presence of a current policy on the effects of the historical Table 1 Fallacy incidence is limited by the fact that the start date of each journal policy is not publicly available. Collectively, these findings suggest that, despite explicit recommendations from multiple journal groups to avoid the Table 1 Fallacy and to adhere to CONSORT guidelines, the most effective strategy for addressing the Table 1 Fallacy may be found in journal-level enforcement.

This study has several limitations that should be carefully considered. The eligibility of trials was based on their registration in ClinicalTrials.gov. As a result, this study may not capture the complete global trials landscape, potentially limiting the generalisability of the findings, and may bias the findings of differential fallacy rates between European versus American journals. It is also important to acknowledge that this study relies on reported data from trials. Trials that conducted testing for baseline differences, but did not include these results in the primary manuscript, could lead to a potential underestimation of the prevalence of the Table 1 Fallacy and introduce bias into associated findings. Finally, while we observed that publication in a European journal was associated with lower odds of the Table 1 Fallacy, this composite journal factor was created following data collection in a post-hoc fashion, and was not an a priori hypothesis at the time of study design; thus, this correlation must be interpreted with caution.

In conclusion, significance testing for baseline covariate differences persists in contemporary randomised controlled trials in oncology. These findings emphasise the need for a collective effort from researchers, the scientific community, and journal editors to address this unproductive and misleading practice and foster a paradigm shift away from baseline significance testing. It is crucial to recognise the inherent limitations and fallacies associated with baseline significance testing in the randomised trial context, and to prioritise education and awareness among trial investigators and peer reviewers. Journal-level enforcement of guidelines and policies may prove to be an effective strategy in mitigating this fallacy.

## Supplementary Material

Supplementary Material

## Figures and Tables

**Table 1:** Characteristics of baseline variable reporting and baseline significance testing in phase III randomised controlled trials in oncology.

	Number of trials (frequency, %)
Number of tabular baseline variables	
1–4	8 (1)
5–9	258 (34)
10–19	418 (55)
20–29	72 (9)
≥30	9 (1)
Significance testing for baseline differences	
Yes	188 (25)
No	577 (75)
Trials with statistically significant tests according to the investigators	
Yes	59 (3)
No	2294 (97)

**Table 2:** Comparison of trials with or without baseline significance testing by univariable binary logistic regression.

		Table 1 Fallacy					
Characteristic	N	Yes, N (%)	No, N (%)	OR	95% CI	*P*	Adjusted *P*^a^
*Total No. of Trials*	765	188 (25%)	577 (75%)				
Disease Stage							
Solid M0	204	64 (31%)	139 (69%)	0.98	0.62–1.55	0.94	1.00
Solid M1	413	77 (19%)	336 (81%)	0.49	0.32–0.75	0.001	0.04
Haematologic	148	47 (32%)	101 (68%)	Ref			
Disease Site							
Breast	146	25 (17%)	121 (83%)	0.39	0.23–0.66	0.0004	0.02
Gastrointestinal	98	18 (18%)	80 (82%)	0.42	0.23–0.77	0.005	0.20
Genitourinary	87	18 (21%)	69 (79%)	0.49	0.27–0.89	0.02	0.80
Haematologic	148	47 (32%)	101 (68%)	0.87	0.55–1.39	0.56	1.00
Thoracic	111	19 (17%)	92 (83%)	0.39	0.22–0.69	0.001	0.04
Other^b^	175	61 (35%)	114 (65%)	Ref			
Treatment Type							
Supportive Care	131	57 (43%)	74 (57%)	3.07	2.06–4.57	<0.0001	<0.0001
Local Therapy	26	9 (35%)	17 (65%)	2.11	0.92–4.85	0.08	1.00
Medical Therapy	608	122 (20%)	486 (80%)	Ref			
Cooperative Group							
Yes	223	92 (41%)	131 (59%)	3.26	2.31–4.61	<0.0001	<0.0001
No	542	96 (18%)	446 (82%)	Ref			
Industry Funded							
Yes	593	103 (17%)	490 (83%)	0.22	0.15–0.31	<0.0001	<0.0001
No	172	85 (49%)	87 (51%)	Ref			
Enrolment^c^							
< 280	191	74 (39%)	117 (61%)	Ref			
< 498	192	44 (23%)	148 (77%)	0.47	0.30–0.73	0.0009	0.04
< 795	190	44 (23%)	146 (77%)	0.48	0.31–0.74	0.001	0.04
≥795	192	26 (14%)	166 (86%)	0.25	0.15–0.41	< 0.0001	< 0.0001
Journal Locale							
European	227	8 (4%)	219 (96%)	0.07	0.03–0.14	<0.0001	<0.0001
American	378	132 (35%)	246 (65%)	Ref			

CI, confidence interval; M0, non-metastatic; M1, metastatic; OR, odd ratio.

a *P* adjusted for multiple comparisons by Bonferroni correction.

b Other disease sites included central nervous system, skin, endocrine, gynaecologic, sarcoma, paediatric, head and neck, and trials evaluating multiple disease sites.

c Categorised by quartiles.

**Table 3:** Factors significantly associated with the Table 1 Fallacy.

Factor of Interest	aOR	95% CI lower	95% CI upper	*P*	Adjusted *P*^a^
*Model 1* (Table S5)					
European journal	0.06	0.03	0.13	<0.0001	<0.0001
American journal	Ref				
*Model 2* (Table S6)					
Enrolment^b^					
< 280	Ref				
< 498	0.55	0.35	0.87	0.01	0.40
< 795	0.60	0.38	0.96	0.03	1.00
≥795	0.32	0.19	0.53	<0.0001	0.0008
*Model 3* (Table S7)					
Industry sponsorship	0.29	0.18	0.47	< 0.0001	< 0.0001

Each model was determined by using structural causal models visualised on directed acyclic graphs to identify confounders for each factor and its relationship to the Table 1 Fallacy. Multivariable binary logistic regressions with Lasso selection to reduce multicollinearity were then performed for each factor and its set of confounders. Bonferroni corrections were applied to each comparison to calculate adjusted *P values. Full models including both factors of interest and confounders are reported in the Supplement*.

CI, confidence interval.

a *P* adjusted for multiple comparisons by Bonferroni correction.

b Categorised by quartiles.

## Data Availability

Research data are stored in an institutional repository and will be shared upon reasonable request to the corresponding author.
