# Supplementary material for: Prevalence and implications of significance testing for baseline covariate imbalance in randomised cancer clinical trials: The Table 1 Fallacy
Source: Eur J Cancer. Author manuscript; Available in PMC 2024 Nov 1. (PMC11512581; doi:10.1016/j.ejca.2023.113357)
Supplement: Supplementary Material [file NIHMS2030930-supplement-Supplementary_Material.docx]

**Supplement**

**Table S1.** Definition of European versus American journal.

**Figure S1**. Flowchart of trial screening and eligibility for analysis.

**Table S2**. Incidence of Table 1 Fallacy by journal.

**Table S3.** Comparison of journal locale with other characteristics.

**Table S4.** Comparison of industry sponsorship with other characteristics.

**Full regression models:**

**Table S5.** European vs American journals.

**Table S6.** Enrollment size

**Table S7.** Industry sponsorship

**Table S8.** Treatment type

**Table S9.** Disease stage

**Table S10.** Disease site

**Table S11.** Cooperative group sponsorship

**Table S1**. Journals defined as either European or American. Journals were required to have published at least 5 of the studied trials to be eligible for this analysis. Journals were also required to have a clearly defined locale or represent a medical society with a clearly defined locale.

| **Journal** | **Medical Society** | **Definition** |
| --- | --- | --- |
| *Lancet* and family | N/A | European |
| *Annals of Oncology* | European Society for Medical Oncology | European |
| *British Journal of Cancer* and family | N/A | European |
| *European Journal of Cancer* | European Organisation for Research and Treatment of Cancer | European |
| *Blood* | American Society of Hematology | American |
| *Cancer* | American Cancer Society | American |
| *International Journal of Radiation Oncology - Biology - Physics* | American Society for Radiation Oncology | American |
| *Journal of the American Medical Association* and family | American Medical Association | American |
| *Journal of Clinical Oncology* | American Society of Clinical Oncology | American |
| *New England Journal of Medicine* | Massachusetts Medical Society | American |

**Figure S1**. Flowchart of trial screening and eligibility for analysis.

**Table S2.** Incidence of the Table 1 Fallacy for all journals publishing at least 5 of the studied trials.

| **Journal** | Published trials, N | Trials with Table 1 Fallacy, N (%) |
| --- | --- | --- |
| *J Clin Oncol* | 196 | 33 (17%) |
| *N Engl J Med* | 128 | 80 (63%) |
| *Lancet Oncol* | 122 | 3 (2%) |
| *Lancet* | 54 | 1 (2%) |
| *Ann Oncol* | 26 | 1 (4%) |
| *Blood* | 17 | 7 (41%) |
| *JAMA* | 15 | 3 (20%) |
| *Support Care Cancer* | 13 | 9 (69%) |
| *Eur J Cancer* | 10 | 1 (10%) |
| *Cancer* | 9 | 3 (33%) |
| *JAMA Oncol* | 8 | 1 (13%) |
| *J Thorac Oncol* | 7 | 1 (14%) |
| *Gynecol Oncol* | 6 | 1 (17%) |
| *Int J Radiat Oncol Biol Phys* | 6 | 6 (100%) |
| *Leukemia* | 6 | 4 (67%) |
| *Breast Cancer Res Treat* | 5 | 0 (0%) |
| *Lung Cancer* | 5 | 1 (20%) |

**Table S3.** Comparison of publication journal locale, when localizable to American vs European, with other studied trial factors.

|  | **Journal Locale** | |
| --- | --- | --- |
| **Characteristic** | **European, N (%)** | **American, N (%)** |
| **Total No. of Trials** | 227 | 378 |
| **Disease Stage** |  |  |
| Solid M0 | 49 (22%) | 102 (27%) |
| Solid M1 | 137 (60%) | 196 (52%) |
| Hematologic | 41 (18%) | 80 (21%) |
| **Disease Site** |  |  |
| Breast | 41 (18%) | 75 (20%) |
| Gastrointestinal | 38 (17%) | 43 (11%) |
| Genitourinary | 22 (10%) | 48 (13%) |
| Hematologic | 41 (18%) | 80 (21%) |
| Thoracic | 50 (22%) | 42 (11%) |
| Other^a^ | 35 (15%) | 90 (24%) |
| **Treatment Type** |  |  |
| Medical Therapy | 201 (89%) | 315 (83%) |
| Supportive Care | 19 (8%) | 48 (13%) |
| Local Therapy | 7 (3%) | 15 (4%) |
| **Cooperative Group** |  |  |
| Yes | 42 (19%) | 136 (36%) |
| No | 185 (82%) | 242 (64%) |
| **Industry Funded** |  |  |
| Yes | 205 (90%) | 278 (74%) |
| No | 22 (10%) | 100 (26%) |

Abbreviations: M0 = non-metastatic; M1 = metastatic.

^a^ Other disease sites included central nervous system, skin, endocrine, gynecologic, sarcoma, pediatric, head and neck, and trials evaluating multiple disease sites.

**Table S4.** Comparison of industry sponsorship with other studied trial factors.

|  | **Industry Funded** | |
| --- | --- | --- |
| **Characteristic** | **Yes, N (%)** | **No, N (%)** |
| **Total No. of Trials** | 593 | 172 |
| **Disease Stage** |  |  |
| Solid M0 | 113 (19%) | 91 (53%) |
| Solid M1 | 362 (61%) | 51 (30%) |
| Hematologic | 118 (20%) | 30 (17%) |
| **Disease Site** |  |  |
| Breast | 105 (18%) | 41 (24%) |
| Gastrointestinal | 84 (14%) | 14 (8%) |
| Genitourinary | 72 (12%) | 15 (9%) |
| Hematologic | 118 (20%) | 30 (17%) |
| Thoracic | 103 (17%) | 8 (5%) |
| Other^a^ | 111 (19%) | 64 (37%) |
| **Treatment Type** |  |  |
| Medical Therapy | 521 (88%) | 87 (51%) |
| Supportive Care | 67 (11%) | 64 (37%) |
| Local Therapy | 5 (1%) | 21 (12%) |
| **Cooperative Group** |  |  |
| Yes | 80 (13%) | 143 (83%) |
| No | 513 (87%) | 29 (17%) |
| **Journal Locale**^b^ |  |  |
| European | 205 (42%) | 22 (18%) |
| American | 278 (58%) | 100 (82%) |

Abbreviations: M0 = non-metastatic; M1 = metastatic.

^a^ Other disease sites included central nervous system, skin, endocrine, gynecologic, sarcoma, pediatric, head and neck, and trials evaluating multiple disease sites.

^b^ If a journal could be localized to a specific region.

**Table S5.** Full multivariable model evaluating the association between European vs American journals and the Table 1 Fallacy.

| **Variable** | **OR** | **95% CI lower** | **95% CI upper** | ***P*** | ***Adjusted P^a^*** |
| --- | --- | --- | --- | --- | --- |
| **Factor of Interest** |  |  |  |  |  |
| European journal | 0.06 | 0.03 | 0.13 | <0.0001 | <0.0001 |
| American journal | Ref |  |  |  |  |
| **Confounder** |  |  |  |  |  |
| Enrollment size^b^ |  |  |  |  |  |
| <280 | Ref |  |  |  |  |
| <498 | 0.57 | 0.31 | 1.04 | 0.07 | 1.00 |
| <795 | 0.55 | 0.30 | 1.00 | 0.05 | 1.00 |
| ≥795 | 0.22 | 0.12 | 0.42 | <0.0001 | 0.0001 |

^a^ *P* adjusted for multiple comparisons by Bonferroni correction.

^b^ Categorized by quartiles.

**Table S6.** Full multivariable model evaluating the association between enrollment size and the Table 1 Fallacy.

| **Variable** | **OR** | **95% CI lower** | **95% CI upper** | ***P*** | **Adjusted *P^a^*** |
| --- | --- | --- | --- | --- | --- |
| **Factor of Interest** |  |  |  |  |  |
| Enrollment^b^ |  |  |  |  |  |
| <280 | Ref |  |  |  |  |
| <498 | 0.55 | 0.35 | 0.87 | 0.01 | 0.40 |
| <795 | 0.60 | 0.38 | 0.96 | 0.03 | 1.00 |
| ≥795 | 0.32 | 0.19 | 0.53 | <0.0001 | 0.0008 |
| **Confounder** |  |  |  |  |  |
| Treatment Type |  |  |  |  |  |
| Supportive Care | 2.41 | 1.58 | 3.66 | <0.0001 | 0.002 |
| Local Therapy | 1.90 | 0.81 | 4.46 | 0.14 | 1.00 |
| Medical Therapy | Ref |  |  |  |  |

^a^ *P* adjusted for multiple comparisons by Bonferroni correction.

^b^ OR reflects enrollment per every 100 subjects.

**Table S7.** Full multivariable model evaluating the association between industry sponsorship and the Table 1 Fallacy.

| **Variable** | **OR** | **95% CI lower** | **95% CI upper** | ***P*** | **Adjusted *P^a^*** |
| --- | --- | --- | --- | --- | --- |
| **Factor of Interest** |  |  |  |  |  |
| Industry sponsorship | 0.29 | 0.18 | 0.47 | <0.0001 | <0.0001 |
| **Confounder** |  |  |  |  |  |
| Cooperative group sponsorship | 1.55 | 0.98 | 2.47 | 0.06 | 1.00 |

^a^ *P* adjusted for multiple comparisons by Bonferroni correction.

**Table S8.** Full model evaluating the association between treatment type and the Table 1 Fallacy.

| **Variable** | **OR** | **95% CI lower** | **95% CI upper** | ***P*** | **Adjusted *P^a^*** |
| --- | --- | --- | --- | --- | --- |
| **Factor of Interest** |  |  |  |  |  |
| Treatment Type |  |  |  |  |  |
| Supportive Care | 2.00 | 1.29 | 3.10 | 0.002 | 0.08 |
| Local Therapy | 0.80 | 0.33 | 1.94 | 0.62 | 1.00 |
| Medical Therapy | Ref |  |  |  |  |
| **Confounders** |  |  |  |  |  |
| Cooperative group sponsorship | 1.63 | 1.02 | 2.61 | 0.04 | 1.00 |
| Industry sponsorship | 0.34 | 0.21 | 0.57 | <0.0001 | 0.001 |

^a^ *P* adjusted for multiple comparisons by Bonferroni correction.

**Table S9.** Full model evaluating the association between disease stage and the Table 1 Fallacy.

| **Variable** | **OR** | **95% CI lower** | **95% CI upper** | ***P*** | **Adjusted *P^a^*** |
| --- | --- | --- | --- | --- | --- |
| **Factor of Interest** |  |  |  |  |  |
| Disease Stage |  |  |  |  |  |
| Solid M0 | 0.98 | 0.62 | 1.55 | 0.94 | 1.00 |
| Solid M1 | 0.49 | 0.32 | 0.75 | 0.001 | 0.04 |
| Hematologic | Ref |  |  |  |  |

Abbreviations: M0 = non-metastatic; M1 = metastatic.

^a^ *P* adjusted for multiple comparisons by Bonferroni correction.

**Table S10.** Full model evaluating the association between disease site and the Table 1 Fallacy.

| **Variable** | **OR** | **95% CI lower** | **95% CI upper** | ***P*** | **Adjusted *P^a^*** |
| --- | --- | --- | --- | --- | --- |
| **Factor of Interest** |  |  |  |  |  |
| Disease site |  |  |  |  |  |
| Breast | 0.39 | 0.23 | 0.66 | 0.0004 | 0.02 |
| Gastrointestinal | 0.42 | 0.23 | 0.77 | 0.005 | 0.20 |
| Genitourinary | 0.49 | 0.27 | 0.89 | 0.02 | 0.80 |
| Hematologic | 0.87 | 0.55 | 1.39 | 0.56 | 1.00 |
| Thoracic | 0.39 | 0.22 | 0.69 | 0.001 | 0.04 |
| Other^b^ | Ref |  |  |  |  |

^a^ *P* adjusted for multiple comparisons by Bonferroni correction.

^b^ Includes central nervous system, skin, endocrine, gynecologic, sarcoma, pediatric, head and neck, and trials evaluating multiple disease sites.

**Table S11.** Full multivariable model evaluating the association between cooperative group sponsorship and the Table 1 Fallacy.

| **Variable** | **OR** | **95% CI lower** | **95% CI upper** | ***P*** | **Adjusted *P^a^*** |
| --- | --- | --- | --- | --- | --- |
| **Factor of Interest** |  |  |  |  |  |
| Cooperative group sponsorship | 1.55 | 0.98 | 2.47 | 0.06 | 1.00 |
| **Confounder** |  |  |  |  |  |
| Industry sponsorship | 0.29 | 0.18 | 0.47 | <0.0001 | <0.0001 |

^a^ *P* adjusted for multiple comparisons by Bonferroni correction.
